# Potential Role of Quercetin Glycosides as Anti-Atherosclerotic Food-Derived Factors for Human Health

**DOI:** 10.3390/antiox12020258

**Published:** 2023-01-23

**Authors:** Junji Terao

**Affiliations:** Faculty of Medicine, Tokushima University, 3-18-15 Kuramoto-cho, Tokushima 770-8503, Japan; terao@tokushima-u.ac.jp

**Keywords:** quercetin glycosides, atherosclerosis, cardiovascular disease, bioavailability, endothelial dysfunction, onion flavonoids, gut microbiota

## Abstract

Quercetin is a monomeric polyphenol of plant origin that belongs to the flavonol-type flavonoid subclass. Extensive studies using cultured cells and experimental model animals have demonstrated the anti-atherosclerotic effects of dietary quercetin in relation to the prevention of cardiovascular disease (CVD). As quercetin is exclusively present in plant-based foods in the form of glycosides, this review focuses on the bioavailability and bioefficacy of quercetin glycosides in relation to vascular health effects. Some glucose-bound glycosides are absorbed from the small intestine after glucuronide/sulfate conjugation. Both conjugated metabolites and deconjugated quercetin aglycones formed by plasma β-glucuronidase activity act as food-derived anti-atherogenic factors by exerting antioxidant, anti-inflammatory, and plasma low-density lipoprotein cholesterol-lowering effects. However, most quercetin glycosides reach the large intestine, where they are subject to gut microbiota-dependent catabolism resulting in deglycosylated aglycone and chain-scission products. These catabolites also affect vascular health after transfer into the circulation. Furthermore, quercetin glycosides may improve gut microbiota profiles. A variety of human cohort studies and intervention studies support the idea that the intake of quercetin glycoside-rich plant foods such as onion helps to prevent CVD. Thus, quercetin glycoside-rich foods offer potential benefits in terms of cardiovascular health and possible clinical applications.

## 1. Introduction

Quercetin (3,3′,4′,5,7-pentahydroxyflavone) is categorized within the flavonol subgroup of flavonoids that is ubiquitously distributed throughout the plant kingdom. Flavonoids are known to participate in plant defense (for example, protection of plants from herbivores, microbial infections, and UV light) and act as attractants for pollinators and seed-dispersing animals, as well as allelopathic agents [1]. Quercetin is frequently found in vegetables, fruits, and cereals in the form of monomeric glycosides, in which various sugars are bound to its hydroxyl groups. Biosynthesis of quercetin starts with the condensation of three malonyl CoA and one *p*-coumaroyl CoA molecule, while quercetin aglycone is formed through successive reactions that produce naringenin–chalcone, naringenin, dihydrokaempferol, and dihydroquercetin [2]. Conversion from aglycone to glycoside is catalyzed by UDP-sugar-dependent glycosyltransferase [3]. Quercetin aglycone acts as a powerful antioxidant because of its ability to scavenge free radicals and chelate transition metal ions [4,5,6,7]. It has been predicted to act as a food-derived physiological antioxidant capable of attenuating oxidative stress, which underlies a variety of degenerative diseases [8,9], in-depth studies into the role of quercetin in the prevention of cardiovascular disease (CVD) strongly suggest that it exerts cardioprotective effects, as oxidative stress is tightly correlated with the onset and progression of CVD [10,11].

CVD is the leading global cause of mortality. In 2019, 17.9 million people died because of CVD, corresponding to 32% of total deaths worldwide [12]. This non-communicable disease includes coronary heart disease (CHD), cerebrovascular disease, and peripheral artery disease. In addition, ischemic heart disease is a type of CVD in which blood flow to heart muscle is reduced or restricted by atherosclerosis. The clinical application of fruits and vegetables has been developed as part of preventive strategies to combat this common chronic disease [13,14]. In 1993, the Zutphen Elderly Study showed for the first time that flavonoid intake from onion, black tea, and apples was inversely associated with mortality from CHD in elderly men [15]. The Seven Countries Study [16] also demonstrated an inverse association between average flavonoid intake and mortality from CHD. Thereafter, large-scale intervention studies, namely the Iowa Women’s Health study [17] and the Danish Cancer and Health cohort study [18] showed that habitual flavonoid intake was inversely associated with all-cause and cardiovascular mortality. Thus, a plentiful intake of dietary flavonoids and flavonoid-rich foods appears to be associated with the prevention of CVD. Recently, numerous in vitro and in vivo studies have proposed a wide variety of mechanisms for the prevention of CVD by flavonoids, such as the improvement of endothelial cell function, suppression of oxidized low-density lipoprotein (oxLDL) accumulation, and anti-inflammatory effects [19,20].

Dietary quercetin appears to act as a powerful preventative factor for CVD by exerting anti-atherosclerotic effects. In plant-based foods, quercetin is mainly present in its glycoside form rather than its aglycone form [21]. This review aims to discuss the significance of the anti-atherosclerotic effects of dietary quercetin glycosides in human health, as well as the prospects of quercetin glycosides as preventive and therapeutic drugs.

## 2. Dietary Intake and Composition of Quercetin Glycosides from Foods

**Figure 1:**
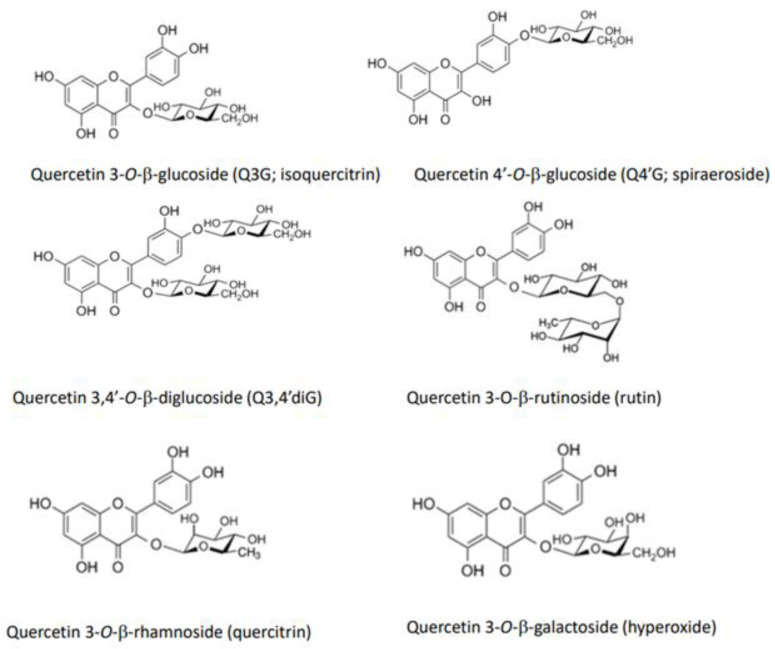
Structures of major quercetin glycosides present in vegetables.

Figure 1 shows the structures of quercetin glycosides, which are mainly present in vegetables. Onion and shallot are examples of vegetables rich in quercetin glycosides, the major components being quercetin 3,4′-*O*-β-diglucoside (Q3,4′diG) and quercetin 4′-*O*-β-glucoside (Q4′G, spiraeoside). These two glucosides, in which the glucose group is bound at the 4′-position, are unique in onion and shallot. In other vegetables, the glucose group is mainly bound at position three in the flavonol structure, forming quercetin 3-*O*-β-glucoside (Q3G, isoquercitrin). Quercetin 3-*O*-β-(6”-malonyl-glucoside) and quercetin 3-*O*-β-glucuronide (miquelianin), quercetin 3-*O*-β-sophoroside (biamaside), and quercetin 3-*O*-β-rutinoside (rutin) are present in lettuce, broccoli, and asparagus, respectively. In addition, onion skin contains considerable amounts of quercetin aglycone, together with Q4′G and Q3,4′diG, although this part of the onion is generally inedible.

Two databases are frequently used to estimate the content of flavonoids in a wide range of foods [22,23]. Table 1 shows the quercetin glycoside content for several vegetables, obtained from the Phenol-Explorer database [22]. In other plant foods, quercetin-3-*O*-β-galactoside (hyperoside) is present in black chokeberry at 46.46 mg/100 g and lingonberry at 13.22 mg/100 g, while Q3G is present in black chokeberry and bottled black tea at 41.95 mg/100 g and 10.87 mg/100 g, respectively. In apple, hyperoside, quercetin-3-*O*-β-alabinoside, and quercetin-3-*O*-β-rhamnoside (quercitrin) are present at 2.36 mg/100 g, 1.40 mg/100 g, and 1.33 mg/100 g, respectively [22]. Furthermore, rutin is found in whole grain buckwheat (36.14 mg/100 g), red raspberry (11.0 mg/100 g), bottled black tea (19.68 mg/100 g), and caper spice (332.29 mg/100 g) [22].

Globally, onion (*Allium cepa* L.) production has reached 44 million tonnes, representing 10% of worldwide vegetable production [24]. According to data provided by the 2002–2004 World Health Survey of fruit and vegetable intake [25], average quercetin intake was estimated at 4.1–5.9 mg/day for groups consuming < 5 servings/day and 9.0–18.0 mg/day for groups consuming > 5 servings/day of fruits and vegetables. Onions accounted for the largest proportion of quercetin intake in all geographic regions.

## 3. Intestinal Absorption and Bioavailability of Dietary Quercetin Glycosides

### 3.1. General Behavior in the Digestive Tract

**Figure 2:**
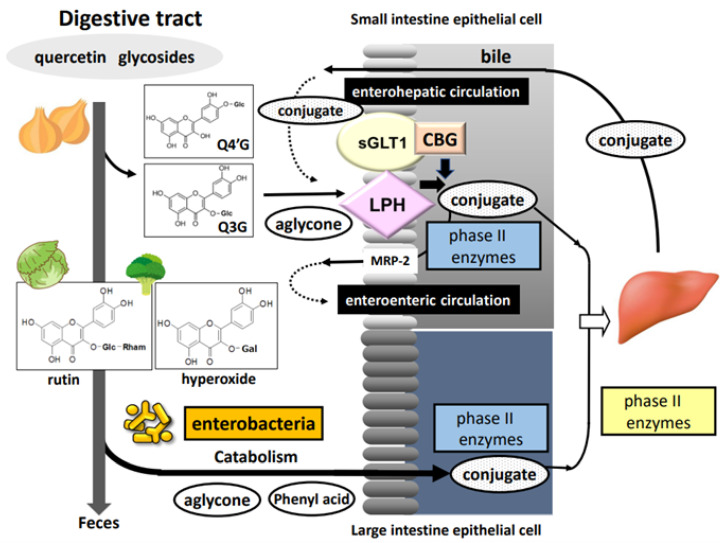
Processing of dietary quercetin glycosides in the intestinal tract (CBG: cellular β-glucosidase; LPH: lactase phlorizin hydrolase; MRP-2: multidrug resistance-associated protein-2; sGLT1: sodium glucose cotranporter-1; Glc: glucose; Gal: galactose; Rham; rhamnose).

Figure 2 shows the general scheme for the processing of quercetin glycosides in the digestive tract. Glucose-bound quercetin glycosides (quercetin glucosides) including Q4′G and Q3G—but not glycosides bound to other sugars, such as rutin, quercitrin, and hyperoside—can be hydrolyzed in the oral cavity by saliva excreted from oral epithelial cells, although hydrolytic activity can vary greatly between individuals [26]. However, most quercetin glycosides reach the small intestine through the esophagus and the stomach. Quercetin glucosides are partially deglycosylated and rapidly absorbed in the small intestine; in addition, conjugation reactions are carried out by phase II drug-metabolizing enzymes present in intestinal epithelial cells [27]. Then, the conjugated metabolites are transported to the liver via the portal route where they undergo secondary metabolism followed by enterohepatic circulation or blood circulation [28]. Conjugated metabolites in the blood circulation are finally excreted into the urine within a few days. In contrast, glycosides bound to sugars other than glucose and unabsorbed quercetin glucosides move to the large intestine, where they are subject to gut microbiota-dependent deglycosylation and degradation reactions, resulting in the production of quercetin aglycone and several types of hydroxyphenyl acid derivatives. Although they are mostly excreted into the feces, some are likely to be absorbed in the large intestine, entering the blood circulation with or without undergoing conjugation reactions, similar to quercetin glucosides in the small intestine [29]. Donovan et al. [30] generalized the pharmacokinetics of quercetin glycosides obtained from different human studies, converting the data to apply to 50 mg doses. Times to reach maximum plasma concentration (*T_max_*) were estimated at 1.5 h and 6 h for quercetin glucosides and rutin, respectively. The longer *T_max_* for rutin is consistent with the absorption of rutin in a more distal part of the intestine following hydrolysis into aglycone by the gut microbiota. In addition, the mean urinary excretion level was calculated to be higher for glucosides (2.5%) than rutin (0.7%).

### 3.2. Absorption and Conjugation of Quercetin Glucosides in the Small Intestine

Quercetin glucosides are deglycosylated to form quercetin aglycone by cellular β-glucosidase (CBG), located in the cytosol of intestinal epithelial cells, and/or lactase phlorizin hydrolase (LPH), a plasma membrane-bound enzyme at the surface [31]. Enzymatic deglycosylation is a critical determinant of bioavailability. Quercetin aglycone produced by the action of LPH is easily absorbed by epithelial cells through passive diffusion because of its enhanced lipophilicity [32]. However, active transport of glucose-bound quercetin glycosides via sodium/glucose cotransporter-1 is required before deglycosylation by CBG in epithelial cells [33].

In the human small intestine, LPH is capable of deglycosylating both Q3G and Q4′G [34]. In contrast, CBG has very little effect on Q3G or Q3,4′diG [35]. Although a preference for deglycosylation of Q4′G rather than Q3G was identified in rat small intestine [36], a previous report [37] demonstrated that the bioavailabilities of Q3G and Q4′G did not differ in humans. Furthermore, a human study showed that Q3,4′diG and Q3G were similarly absorbed and transferred into the bloodstream [38]. Q3,4′diG appears to be absorbed into the body after simultaneous deglycosylation of the two glucose-bound groups at the 3- and 4′-positions by LPH or after Q4′G formation by LPH, followed by complete deglycosylation by CBG to release quercetin aglycone.

Neither hydrolyzing enzyme acts on glycosides bounds to sugars other than glucose, resulting in little absorption of such glycosides in the small intestine [39]. A study in rats suggested that rutin was absorbed more slowly than quercetin aglycone because of a requirement for deglycosylation by the cecal microbiota [40,41]. In addition, a human study with 15 subjects showed that the level of excretion of unmodified quercetin was 0.5% of intake after the consumption of strong black tea and 1.1% after the consumption of onions [42]. The lower level of excretion following tea intake may have resulted from the lower efficiency of intestinal absorption of rutin (present in tea), compared with the absorption of quercetin glucosides (present in onion). However, Erlund et al. [43] reported that the mean area under the curve (AUC) for plasma concentration–time and the maximum plasma concentration (*C_max_*) of the two compounds were similar, although the *T_max_* was significantly shorter after quercetin aglycone treatment than after rutin treatment. Jaganath et al. [44] demonstrated that rutin was mostly absorbed in the large intestine by comparing the metabolic profiles of healthy volunteers and ileostomy patients who had ingested rutin.

### 3.3. Metabolism of Quercetin Glucosides and Circulation of Their Metabolites

During intestinal absorption, quercetin glucosides are converted to their conjugated metabolites, glucuronides and sulfates, by UDP-glucuronosyl transferase and phenol sulfate transferase, respectively. The phenol groups of these conjugates can also be subject to *O*-methylation by the action of catechol-*O*-methyl transferase [45]. These conjugated metabolites are further metabolized to produce more complex compounds in the liver after transfer via the portal route. Conjugated metabolites derived from quercetin glucosides have also been suggested to be partly transferred to the blood circulation via the lymphatics [46]. The enterohepatic circulation of quercetin metabolites between the liver and the digestive tract via the gallbladder may affect their bioavailability as a result of reabsorption in the digestive tract. Epithelial multidrug resistance-associated protein 2 plays a role in the enteroenteric circulation of conjugated metabolites and modulation of quercetin glucoside bioavailability.

**Figure 3:**
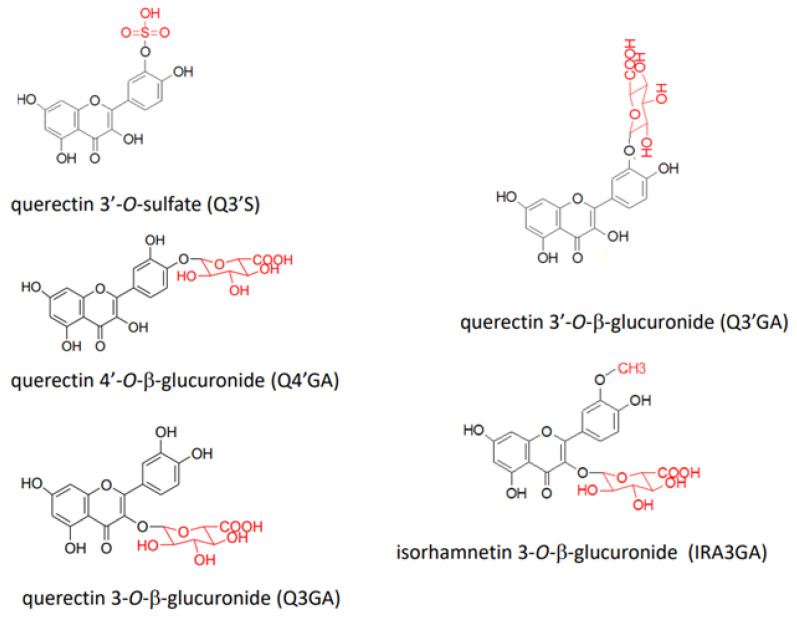
Structures of representative quercetin conjugated metabolites detected in human plasma.

In a human study, 23 species of conjugated metabolites, including quercetin 3-*O*-β-glucuronide (Q3GA), quercetin 3′-*O*-β-glucuronide (Q3′GA), quercetin 4′-*O*-β-glucuronide (Q4′GA), quercetin 3′-*O*-sulfate (Q3′S), and isorhamnetin 3-O-β-glucuronide (IR3GA), were identified in the plasma 1 h to 4 h after the intake of red onion (Figure 3) [47]. In addition, comparing human plasma after the consumption of onion powder containing Q4′G and Q3,4′diG or apple skin containing quercetin-3-*O*-arabinoside, hyperoside, Q3G, and quercitrin revealed that similar conjugated metabolites (identified as quercetin sulfate, quercetin glucuronide, and quercetin diglucuronide) were produced, although their relative ratios differed [48,49]. Using physiologically based kinetic modeling to predict the plasma concentration of flavonoids, Boonpawa et al. [50] suggested that Q3′GA was the major circulating metabolite in 19 out of 20 individuals.

### 3.4. Catabolism of Quercetin Glycosides by the Microbiota in the Large Intestine

**Figure 4:**
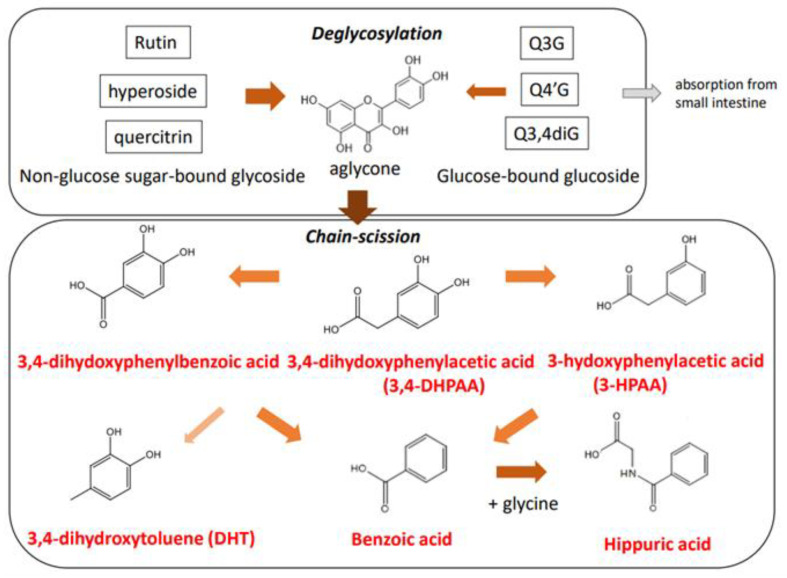
Gut microbiota-dependent catabolic pathway of quercetin glycosides in the large intestine.

In the large intestine, quercetin glycosides are subject to enzymatic ring-fission reactions and deglycosylation by the gut microbiota (Figure 4). Although the products are mostly excreted into the feces, a portion can reach the circulation following absorption in the large intestine. The gut microbiota can convert rutin to phenyl acid derivatives, namely 3-hydroxy phenylacetic acid (3HPAA) (36%), 3-methoxy-4-hydroxyphenylacetic acid (8%), and 3,4-dihydroxyphenylacetic acid (3,4-DHPAA) (5%) [51]. Degradation of quercetin aglycone by *Eubacterium ramulus*, a strict anaerobe resident in the human intestinal tract, also produces 3,4-DHPAA [52]. Furthermore 3,4-DHPAA and 4-hydroxybenzoic acid, in addition to quercetin aglycone, were detected as catabolites following the anaerobic incubation of quercitrin with human intestinal bacteria [53]. In the case of rutin catabolism by human fecal bacteria, deglycosylation to produce quercetin aglycone and subsequent degradation to form hydroxyphenyl acid derivatives depend on interindividual gut microbiota composition [54]. In addition, the catabolic profile of hyperoside produced by human intestinal bacteria revealed six metabolites, including quercetin aglycone, 3,4-DHPAA, and 3,4-dihydroxyphenylbenzoic acid [55].

These degradation products are suggested to enhance intestinal health by modulating direct or indirect immune functions such as T-cell differentiation and gut microbiota turnover [56]. Hydroxyphenyl acid derivatives are also expected to exert their effects in the blood circulation after transfer from the large intestine. However, little is known about absorption mechanisms in the large intestine. Nonetheless, these hydroxyphenyl acid derivatives and their conjugated metabolites appear to be present in human plasma following the intake of quercetin glycosides, as demonstrated for hesperetin-7-rutinoside and naringenin 7-rutinoside after the ingestion of orange juice [57].

## 4. Modulation of Bioavailability

### 4.1. General Comments

The bioavailability of quercetin glycosides is highly influenced by the type and amount ingested. Interindividual differences in intestinal deglycosylation and absorption efficiencies and microbiota profiles are also endogenous determinants of quercetin glycoside bioavailability [58]. In a human study whereby large amounts of quercetin aglycone (500 mg/day or 1000 mg/day) were used as dietary supplements for 12 weeks, the overnight-fasted plasma concentrations of quercetin metabolites were significantly increased. Although the increases were highly variable between individuals, they were not related to gender, BMI, fitness level, or dietary intake [59]. Nevertheless, several strategies aimed at improving the bioavailability of quercetin glycosides have been applied to better realize the cardioprotective activities of these compounds in vivo [60].

### 4.2. Effects of the Food Matrix

The food matrix has been shown to increase the bioavailability of quercetin aglycone. For example, a randomized, single-blind, diet-controlled study demonstrated that for equivalent intakes of quercetin (130 mg), quercetin-enriched cereal bars were more effective at increasing the plasma levels of quercetin metabolites than quercetin powder-filled capsules [61]. In addition, comparison of AUC and *C_max_* following the ingestion of quercetin aglycone derived from onion skin extract powder (163 mg) or pure quercetin dihydrate (136 mg) showed that the values were 4.8-fold and 5.4-fold higher, respectively, for the onion skin extract [62]. Thus, the amorphous structure of quercetin aglycone from onion skin extract appears to improve solubility in the intestinal tract, in comparison with the crystal structure of quercetin dihydrate.

A cross-over trial was carried out to compare the bioavailability of quercetin administered in the form of fresh red onion (quercetin glycosides: 47 mg quercetin aglycone equivalent) with that of quercetin in the form of a dietary supplement (quercetin aglycone tablet: 544 mg quercetin aglycone equivalent) [63]. The results showed that the 24-h urinary excretion levels of total quercetin following the consumption of onion (1.69 ± 0.79 μmol) or quercetin supplement tablets (1.17 ± 0.44 μmol) were not significantly different. These findings indicated that quercetin aglycone was more efficiently available from onions than from quercetin supplements. The food matrix is likely to contribute to these differences in bioavailability. In addition, Wiczkowski et al. [64] compared the bioavailability of 10 mg quercetin aglycone equivalent in dry shallot skin (containing quercetin aglycone at 83.3% and quercetin glucosides at 16.7%) and shallot flesh (containing quercetin glucosides at 99.2% at intake). The results showed that the AUC value for intake via dry shallot skin was more than twice as high as the value for shallot flesh, indicating that the bioavailability of quercetin aglycone was dependent on dietary source in humans. It is therefore clear that the lower bioavailability of quercetin aglycone, compared with quercetin glucosides, is caused by the lower solubility of pure quercetin aglycone (supplied in the form of tablets or capsules) in the digestive tract. In addition, a human ingestion study confirmed that onion peel powder performed better than onion peel extract at increasing the plasma concentration of quercetin [65]. Thus, the food matrix acts as a key factor for improving the bioavailability of quercetin in either aglycone or glucoside form. Therefore, lower levels of quercetin ingested from foods may be as effective as higher levels of pure quercetin ingested in the form of supplements at inducing the vascular functions of this compound.

### 4.3. Effect of Coexisting Ingredients

Studies have shown that dietary fat increases the micellization efficiency of quercetin aglycone, improving its intestinal absorption. For example, a randomized cross-over trial in overweight adults showed that the inclusion of 15.4 g of fat alongside 1095 mg quercetin aglycone resulted in increases of 45% and 32% in the *C_max_* and AUC values, respectively [66]. Moreover, a study conducted in rats demonstrated that the addition of more than 4.6% of soybean oil to an onion powder-containing diet for one or two weeks enhanced the plasma concentration of quercetin [67]. Therefore, it is likely that fat and oil increase the absorption of quercetin glucosides in the intestinal tract by assisting micellization, regardless of supplementary or dietary source. However, the effect of fat on the bioavailability of quercetin glucosides in humans is unknown. In addition, combining cooked sweet potato with fried onion was found to suppress increases in plasma quercetin metabolite concentrations in humans. This suppressive effect was probably caused by excess carbohydrate digestion products from the sweet potato interfering with quercetin glucoside deglycosylation during intestinal epithelial uptake [68]. Combining onion and tofu also affected the plasma profiles of quercetin metabolites, with quercetin sulfate only detected after combined intake [69]. Therefore, specific food combinations may considerably affect the bioavailability and metabolic profiles of quercetin glucosides after dietary ingestion.

### 4.4. Effects of Cooking and Processing

Cooking lowered the quercetin content of tomatoes and onions, with greater reductions caused by microwaving and boiling than by frying [70]. Ioku et al. [71] demonstrated that microwaving and frying did not affect the content of quercetin glucosides in onion flesh, although boiling led to a loss of approximately 30% because of transfer to the boiling water. The effects of food processing (including mechanical processing, thermal and non-thermal treatments, oral delivery and nanoformulations, enzymatic hydrolysis, and fermentation) on the bioavailability of flavonoids have been extensively reviewed by Polia et al. [72]. In addition, to increase water solubility, quercetin glucoside can be enzymatically modified to produce isoquercitrin derivatives containing 1–6 glucose moieties at the 3-glucoside position of Q3G (EMIQ). In a human study with 100 mg quercetin equivalent intake, EMIQ produced a higher plasma concentration of quercetin conjugates 90 min after ingestion, compared with intact Q3G [38]. EMIQ appears to be effectively transported to intestinal epithelial cells through the “unstirred” water layer before being hydrolyzed by mucosal maltase–glucoamylase and LPH and efficiently incorporated into cells as aglycone.

## 5. Mechanisms of Onset of Atherosclerosis

**Figure 5:**
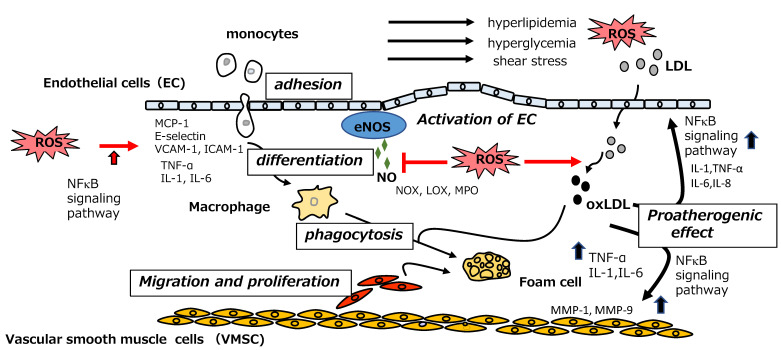
Vascular events in the initial stage of atherosclerosis (eNOS: endothelial nitric oxide synthase; ICAM-1: intermolecular adhesion molecule-1; IL-1; interleukin-1, IL-6: interleukin-6; IL-8: interleukin-8; LDL; low-density lipoprotein; LOX; lipoxygenase; MCP-1: monocyte chemotactic protein-1; MMP-1: matrix metalloproteinase-1; MMP-9: matrix metalloproteinase-9; MPO: myeloperoxidase; NFκB: nuclear factor kappa B; NOX; NADPH oxygenase; oxLDL: oxidized low-density lipoprotein; ROS: reactive oxygen species; TNF-α: tumor necrosis factor-α; VCAM-1: vascular cell adhesion molecule-1).

Atherosclerosis is a potentially serious condition whereby arteries become clogged with fatty streaks termed plaques or atheroma. The resulting constriction and injury of vessel lumens cause a deficiency in the supply of oxygen from the blood to the heart, damaging the cardiac muscle and leading to CVD. Arterial blood vessels are composed of adventitia, media, and intima. The surface of the intima is covered by the vascular endothelium, which is formed of a monolayer of endothelial cells. Endothelium represents the first barrier encountered by molecules, cells, and pathogens circulating in the bloodstream and performs a wide variety of vascular functions including control of vascular permeability, vasoconstriction, vasodilation, and thrombus formation. The steps that lead to the development of atherosclerosis are described below [73,74] (see Figure 5).

### 5.1. Endothelial Dysfunction and Formation of oxLDL

Endothelial cells act as sensors for mechanical stress on the vascular wall (wall shear stress, WSS) caused by disturbances in blood flow. They also act as signal transducers through the production of biologically active substances, inducing the expression of monocyte chemotactic protein-1 (MCP-1) and the proliferation of vascular smooth muscle cells (VSMCs) in the vascular media. Nitric oxide (NO) produced by endothelial NO synthase (eNOS) acts as an endothelially-derived vasodilator, exerting an anti-atherosclerotic effect. However, NO is deactivated by the overproduction of reactive oxygen species (ROS) under conditions of oxidative stress such as hyperlipidemia, hypertension, and diabetes. Furthermore, oxidative stress leads to the production of pro-inflammatory cytokines including tumor necrosis factor-α (TNF-α), interleukin-1β (IL-1β), and interleukin-6 (IL-6), as well as the upregulation of adhesion molecules such as vascular cell adhesion molecule-1 (VCAM-1) and intermolecular adhesion molecule-1 (ICAM-1), through activation of the nuclear factor kappa B (NFκB) signaling pathway.

WSS distorts the structure of the vascular endothelium, leading to the subendothelial infiltration of plasma LDL via caveolae-dependent transcytosis. In addition, the activation of endothelial cells by inflammatory cytokines results in the recruitment of monocytes to the endothelium. After migrating to the endothelial cell surface, monocytes are subjected to rolling by E-selectin molecules and firm attachment to the endothelium through interactions with VCAM-1 and ICAM-1. Then, monocytes infiltrate the intima via paracellular or transcellular processes. Once within the intima, monocytes differentiate into macrophages, which can be polarized to M1 (pro-inflammatory) or M2 (anti-inflammatory) phenotypes; atherosclerotic conditions favor M1 polarization. M1 macrophages release the inflammatory cytokines IL-1β, IL-6, and TNF-α, which promote monocyte recruitment and propagate the inflammatory response. Infiltrated LDL particles are converted to oxLDL by oxidative modification of the tyrosine residue of apolipoprotein B-100. Myeloperoxidase (MPO), 15-lipoxygenase, and NADPH oxygenase (NOX), which are activated by endothelial dysfunction, are suggested to be responsible for this oxidative modification.

### 5.2. Phagocytosis of oxLDL by Monocyte-Derived Macrophages and Foam Cell Formation

Monocyte-derived macrophages internalize oxLDL by phagocytosis through LDL scavenger receptors, CD36, scavenger receptor A1 (SRA-1), and oxLDL receptor (lectin-like oxidized LDL receptor-1, LOX-1). The accumulation of oxLDL within macrophages leads to foam cell formation. Following the lysosomal degradation of oxLDL, the released cholesterol is esterified by acyl-CoA cholesterol acyltransferase at the endoplasmic reticulum and distributed as droplets in the cytosol. Next, free cholesterol hydrolyzed from esterified cholesterol by the action of cholesteryl ester hydrolase is transferred into high-density lipoprotein (HDL) via ABC transporter A1 (ABCA1) and ABC transporter G1 (ABCG1). This event is an essential step for the recovery of excess cholesterol from peripheral tissue and an important process in the prevention of atherosclerosis. However, under inflammatory conditions, the excretion of excess cholesterol by ABC transporters does not occur, thus promoting foam cell formation.

### 5.3. Proatherogenic Effect of oxLDL on Endothelial Cells and VSMCs

oxLDL activates the NFκB signaling pathway in macrophages, resulting in the production of inflammatory cytokines, activation of endothelial cells, monocyte infiltration, and, finally, foam cell formation. In addition, the uptake of oxLDL by endothelial cells via LOX-1 accelerates endothelial dysfunction by inducing the production of pro-inflammatory cytokines. Furthermore, unrestricted uptake of oxLDL by VSMCs occurs via the receptors SRA-1, CD-36, and LOX-1, leading to foam cell formation. Once taken up by VMSCs, oxLDL induces the expression of the collagen-decomposing enzymes matrix metalloproteinase-1 and matrix-metalloproteinase-9, resulting in the degradation of extracellular collagen, which finally leads to destabilization of the foam cells.

### 5.4. Fibrosis of Blood Vessels and Thrombus Formation

Elevation of plasma LDL levels by hypercholesterolemia leads to continual repetition of the above processes. Atheroma (fibrous plaques) develops gradually, and the arteries become narrow and hard. Decreased vessel flexibility results in atherosclerosis and the induction of hypertension. Hypertrophied plaques can become vulnerable, and then thrombosis occurs in the vessel because of plaque disruption, resulting in the blockage of blood flow.

## 6. Proposed Mechanism for CVD Prevention by Quercetin Glycosides

Scavenging of oxidative stress-inducing free radicals by the phenolic hydroxyl groups of quercetin and its glycosides enables these compounds to act as powerful antioxidants. However, it is worth pointing out that the bioavailability of quercetin glycosides appears to be lower than that of antioxidant vitamins such as vitamin C and vitamin E. Indeed, an essential mechanism of the anti-atherosclerotic effects of quercetin and its glycosides appears to be indirect antioxidant activity. That is, these compounds modulate the activity and expression of oxidative/antioxidative enzymes responsible for the regulation of oxidative stress, attenuating inflammatory activity by suppressing the production of pro-inflammatory enzymes and cytokines [74,75].

### 6.1. Mechanism of Anti-Inflammatory Activity of Phenolic Compounds

A series of anti-inflammatory functions performed by phenolic compounds (summarized in a review by Rhaman et al. [76]) is outlined below.

- Phenolic compounds inhibit the NFκB signaling pathway, resulting in downregulated gene expression of inflammation-inducing enzymes such as cyclooxygenase-2 (COX-2), inducible NOS (iNOS), and 5-lipoxygenase (5-LOX) and inflammatory cytokines such as IL-1β, IL-6, and TNF-α.
- Phenolic compounds prevent the adhesion of monocytes to endothelial cells by suppressing the expression of E-selectin, MCP-1, VCAM-1, and ICAM-1.
- Phenolic compounds attenuate endothelial oxidative stress by inhibiting NOX-dependent ROS production. They also elevate levels of the endothelial anti-atherosclerotic factor NO by accelerating the phosphorylation of eNOS via an AMP-activated protein kinase (AMPK)-dependent mechanism. In addition, phenolic compounds elevate eNOS expression by activating the nuclear factor E2-related factor 2 (Nrf2) pathway.
- Phenolic compounds suppress the migration of VSMCs by inhibiting platelet-derived growth factor-related signaling molecules such as phosphoinositide-3-kinase (PI3K). They also suppress VSMC growth by inducing apoptosis via activation of p38 mitogen-activated protein kinase and P53 signaling pathways.

In vitro cultured cell experiments showed that quercetin aglycone inhibited the generation of ROS by COX-2 and 5-LOX, downregulated the production of COX-2 and NFκB, and suppressed the secretion of IL-6 and TNF-α by lipopolysaccharide (LPS)-stimulated macrophages [77]. Quercetin aglycone also inhibited the secretion of IL-1β, IL-6, IFN-γ, and TNF-α by LPS-stimulated blood cells [77]. Moreover, quercetin aglycone was reported to suppress the production of NO, IL-6, MCP-1, TNF-α, iNOS, and COX-2 by downregulating the NFκB signaling pathway [77] and to exert anti-inflammatory effects by inhibiting tyrosine kinase, Src, and Syk-mediated PI3K phosphorylation and expression of VCAM-1 and CD80 [78]. Activation of the Nrf-2 signaling pathway may contribute to the suppression of ICAM-1 and E-selectin [79].

### 6.2. Insights from In Vivo Animal Experiments

#### 6.2.1. Modulation of Inflammatory Cytokines and Oxidative Stress

Carrageenan-induced inflammation in rats under oxidative stress was found to be reduced by pretreatment with 10 mg/kg body weight (BW) of quercetin aglycone, isoquercitrin, or rutin, which inhibited prostanoid synthesis and cytokine production [80]. In addition, oral administration of quercetin aglycone (five doses of 150 mg/kg BW) resulted in reduced clinical signs in an experimental rat model of arthritis, correlating with decreased levels of macrophage inflammatory mediators [81]. In rats with experimental autoimmune myocarditis, quercetin aglycone administered at a dose of 20 mg/kg BW for 21 days reduced clinical symptoms and serum levels of TNF-α and IL-17 while increasing IL-10 levels [82]. Furthermore, the intraperitoneal injection of quercetin aglycone (100–200 mg/kg BW) in a hypertriglyceridemia-related rat model of acute pancreatitis led to attenuated pancreatic histopathological damage and decreased mRNA and protein expression of NF-κB, IL-1β, IL-6, and TNFα [83].

Both quercetin aglycone and rutin partially but significantly attenuated lipid peroxidation in normal and diabetic model rats with ischemia-reperfusion injury, suggestive of cardioprotective effects [84]. Meanwhile, quercetin treatment (50 mg/kg BW) was found to enhance the activity of Cu/Zu-superoxide dismutase (SOD) and glutathione peroxidase and reduce the level of lipid peroxidation products in brain edema induced in an experimental rat model of subarachnoid hemorrhage [85]. Furthermore, administration of quercetin (50–150 mg/kg BW) for 30 days significantly ameliorated myocardial oxidative injury and immune function impairment in a rat model of myocardial ischemia [86]. In the same ischemia model, the cardioprotective effect of rutin was found to be weaker than that of quercetin aglycone at the same dose, despite the cardioprotective effects of both phenolic compounds in cardiac fibrosis [87].

#### 6.2.2. Modulation of Plasma LDL Levels

Elevation of plasma LDL levels is one of the risk factors for atherosclerosis. In terms of atherosclerosis prevention, quercetin and its glycosides have generated interest because of their potential LDL-lowering effects during various processes, including intestinal cholesterol absorption, very-low-density lipoprotein assembly, LDL metabolism and endocytosis, and bile acid metabolism [88]. For example, quercetin aglycone has been shown to influence the activity of the sterol transporter protein Nieman-Pick C1-like 1 (NPC1L1). Quercetin aglycone decreased serum cholesterol levels when administered alongside a high-cholesterol diet, likely because of its specific inhibition of intestinal cholesterol absorption mediated by NPC1L1 [89].

Several in vivo experiments have demonstrated the effects of dietary quercetin glucosides administered to animals on high-cholesterol diets. For example, New Zealand white rabbits were maintained on a 2.0% cholesterol diet with 0.1% Q3G for a month [90]. Triacylglycerol and cholesterol levels in the plasma and aorta were lowered and lipid peroxidation biomarkers were decreased by the co-administration of Q3G, indicating that Q3G attenuated lipid peroxidation in the aorta, as well as suppressing hyperlipidemia. In mice fed a 1.0% cholesterol diet for 12 weeks, supplementation with Q3G (0.05%–0.1%) led to the attenuation of hyperinsulinemia, as determined by decreased plasma levels of hepatic LDL receptor and proprotein convertase subtilisin kexin 9 (PCSK9), a regulator of plasma LDL cholesterol clearance [91]. Additionally, in apo-E^−/−^ mice fed a high-fat diet for 12 weeks, plaque formation and levels of PCSK9, TNF-α, and IL-6 were significantly reduced by co-administration of quercetin aglycone at 12.5 mg/kg BW; meanwhile, the expression of ABCA1 and anti-atherosclerotic liver X receptor-α (LXR-α) was significantly increased [92].

Liver cholesterol is converted to bile acid by the action of the drug-metabolizing enzymes CYP7A1, CYP27A1, and CYP7B. Bile acid is released into the duodenum via the bile duct and reabsorbed by intestinal epithelial cells via apical sodium-dependent bile acid transporter (ASBT) and ileal bile acid-binding protein. Bile acid is then subject to reuptake into the liver by sodium-dependent taurocholate co-transporting peptide in a recycling process known as enterohepatic circulation. Therefore, increased excretion of bile acid leads to accelerated bile acid synthesis. Quercetin has been suggested to accelerate bile acid synthesis by upregulating CYP7A1 expression, resulting in lower cholesterol efflux. For example, in a rat study, a quercetin-based diet increased expression of CYP7A1, LXR-α, and ABCG1 [93]. In a different study carried out in mice, dietary supplementation with black bean extract containing Q3G resulted in elevated CYP7A1 expression and increased excretion of bile acid and cholesterol via bile acid synthesis [94]. Quercetin may also decrease plasma LDL levels by downregulating the expression of ASBT, which is responsible for normal enterohepatic circulation [95]. These reports indicate that quercetin has the potential to exert anti-atherosclerotic effects by regulating plasma LDL concentration.

### 6.3. In Vivo Animal Studies on the Anti-Atherogenic Effects of Onion Intake

Onions have been reported to protect against experimentally induced atherosclerosis. For example, in rats fed on a high-fat diet for 8 weeks, co-administration of onion peel extract (0.2% in the diet) increased ABCA1 expression, lowered LDL levels, and increased HDL levels [96]. In another study, co-administration of 10% onion powder to rats on a high-cholesterol diet (2%) for 7 weeks resulted in increased antioxidant activity in mesenteric microvessels, with the upregulation of SOD and GPX activity and the suppression of NOX activity [97]. Thus, enriching the diet with onion as a functional ingredient is proposed to prevent the vascular dysfunction associated with the initial development of atherosclerosis. In the same experimental system, onion powder increased anti-inflammatory ω-3 oxylipins levels in the liver, thus apparently exerting anti-inflammatory effects through the regulation of pro-inflammatory mediators [98]. Including onion in the diet of rats has also been found to influence the levels of specific primary and secondary bile acids, indicating that onion acts as a critical modifier of fecal bile acid composition linked to hypercholesterolemia [99]. In addition, administration of onion peel extract at 200 mg/kg BW to mice maintained on a 1.25% cholesterol diet for 12 weeks resulted in decreased LDL cholesterol levels, lower atherogenic index values, and concomitant increases in LDL receptor and CYP7A1 mRNA levels; these findings indicated that onion intake lowered cholesterol in the plasma and liver by inducing the excretion of cholesterol into the feces [100]. In a similar experiment, intake of onion extract (2.42 mg/kg BW quercetin aglycone plus 4.60 mg/kg BW Q4′G) ameliorated hyperlipidemia in rats by upregulating liver LDL receptor and downregulating 3-hydroxy-3-methylglutaryl-CoA reductase, an essential enzyme for cholesterol synthesis [101]. Nevertheless, it should be noted that active components other than quercetin aglycone and its glycosides may be responsible (independently or synergistically) for the cardioprotective effects of such extracts.

## 7. Disadvantages of In Vitro Cultured Cell and In Vivo Animal Studies for Elucidating the Mechanism of Action of Quercetin Glycosides

Quercetin glycosides are suggested to exert antioxidant and anti-inflammatory effects by modulating the activities of inflammation-related enzymes and cytokines directly, at the target site, or indirectly, through the regulation of gene expression via specific cellular signaling pathways. However, our mechanistic understanding of the cardioprotective effects of quercetin glycosides remains poor, as individual reports often fail to provide a comprehensive overview of potential integrative mechanisms. In addition, unphysiologically-high concentrations of quercetin compounds (>μM) have frequently been used in cultured cell studies as well as animal studies, which may not be appropriate for disease prevention in humans. So far, it is unknown to what extent the mechanisms proposed in these studies are responsible for the prevention of CVD in humans. It should be emphasized that neither quercetin aglycone nor quercetin glycosides are easily absorbed into the body, and even after absorption, they are mostly present in the form of conjugated metabolites in the blood. Therefore, the functions of these conjugated metabolites should be clarified in cultured cell studies to fully explore their protective mechanisms.

### 7.1. Mechanisms of Action of Conjugated Metabolites

#### 7.1.1. Modulation of Oxidative Enzymes and Inflammatory Enzymes

Quercetin glucuronides have been found to exert inhibitory effects on xanthin oxidase (XOD) and LOX at expected plasma concentrations, depending on the conjugation position [102]. Q3′S retained considerable LOX inhibitory activity, while Q3GA had no LOX inhibitory activity [103]. Nonetheless, Q3GA was found to act as an effective inhibitor of MPO, an inflammatory enzyme secreted by activated neutrophils and macrophages [104,105]. Another study showed that quercetin plasma metabolites, including Q3GA, inhibited COX-2 mRNA expression in human lymphocytes ex vivo, although, in vivo, a single high dose of quercetin glucosides from onions had no effect on COX-2 mRNA levels [106].

#### 7.1.2. Protection of Endothelial Function

Quercetin plasma metabolites including Q3GA and IR3GA induced the activation of AMPK and eNOS in human aortic endothelial cells (HAECs), indicating that these metabolites may protect endothelial function in part via the AMPK pathway [107]. Q3GA also ameliorated endothelial insulin resistance by suppressing excess ROS-induced inflammation via inhibition of IL-6 and TNF-α production [108]. However, Q3GA did not suppress TNF-α-induced ICAM-1 expression in IL-1α-stimulated HAECs, despite elevated permeability [109]. Furthermore, although Q3′S, Q3GA, and IR3GA suppressed the recruitment of monocytes to endothelial cells by inhibiting the cell-surface expression of VCAM-1 [110], neither Q3GA nor Q3′S affected the expression of cell adhesion molecules in LPS-stimulated human neutrophils [111].

Caveolin-1 plays a key role in endothelial dysfunction, acting as a platform for proatherogenic signal transduction in the caveolar membrane of endothelial cells [112]. Physiological concentrations (1 μM) of quercetin aglycone and Q3GA were equally effective at inhibiting oxLDL-induced expression of caveolin-1; both compounds also downregulated adhesion molecule expression in human umbilical vein endothelial cells (HUVECs) [113]. However, Q3GA did not inhibit the hydrogen peroxide-induced phosphorylation of caveolin-1, which increases the permeability of endothelial cells [114].

#### 7.1.3. VSMCs as Cellular Targets

Neither Q3GA, Q3′-S, nor IR3GA affected the expression of VCAM-1, ICAM-1, or MCP-1 in TNF-α-stimulated human umbilical artery smooth muscle cells (HUASMCs) [115], suggesting that the effects of conjugated metabolites in blood vessels are not mediated via VSMCs. Nevertheless, Q3GA was found to suppress angiotensin II-induced c-Jun-N-terminal kinase (JNK) activation, thereby inhibiting DNA binding by activator protein-1 (AP-1) and preventing rat aortic smooth muscle cell hypertrophy [116]. Q3GA also suppressed platelet-derived growth factor-induced cell migration and proliferation by inhibiting the JNK and AP-1 signaling pathways [117].

#### 7.1.4. Vasodilation and Vasoconstriction

Q3′S but not Q3GA was shown to inhibit endothelin-induced contraction of porcine coronary artery segments [118]. In addition, a HUASMC and HUVEC co-culture model study confirmed the beneficial effects of conjugated metabolites of quercetin on vascular function [119].

### 7.2. Deconjugation of Quercetin Glucuronides

**Figure 6:**
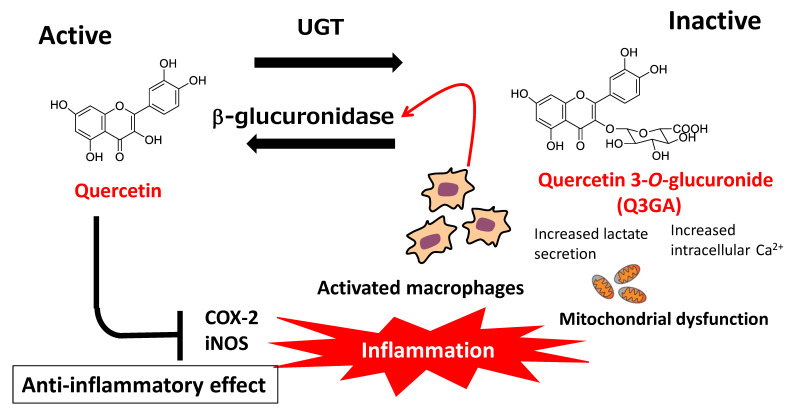
Physiological significance of deconjugation of quercetin conjugated metabolites: conjugation–deconjugation cycle (COX-2: cyclooxyganse-2; iNOS: inducible nitric oxide synthase; UGT: UDP glucuronosyl transferase).

As described above, quercetin is mainly present in the blood circulation in the form of hydrophilic conjugated metabolites. In general, conjugation reactions constitute a detoxification process for xenobiotics, as well as an inactivation process for dietary flavonoids. However, the anti-inflammatory effects of conjugated quercetin glucuronides have been suggested to result from deconjugation reactions that occur during inflammation and produce the parent aglycone [120,121]. Shimoi et al. [122] first reported that a flavone-type flavonoid, luteolin, was deconjugated by stimulated human neutrophils, while β-glucuronidase activity was increased in the plasma of LPS-injected rats and serum of patients undergoing hemodialysis. These findings indicated that the deconjugation of flavonoid metabolites was carried out in the plasma by β-glucuronidase, derived from stimulated neutrophils under inflammatory conditions. Kawai et al. [123] demonstrated the uptake of Q3GA by activated macrophages in human atherosclerotic lesions and showed that the anti-inflammatory effects of Q3GA were mediated by the release of its aglycone. In addition, they found that the extracellular efflux of β-glucuronidase from macrophages was triggered by increased levels of cellular calcium ions caused by mitochondrial dysfunction, while acidification around the cellular surface was also necessary for efficient enzyme activity [124]. The effects of activated macrophages on deconjugated quercetin aglycone levels represent a plausible explanation for the anti-atherosclerotic effects of dietary quercetin glycosides, which are characterized by low bioavailability and high susceptibility to metabolic conversion (Figure 6).

Further experiments showed that when Q3GA was perfused through isolated rat mesenteric vascular beds, Q3GA was slowly deconjugated to its aglycone in the perfusate and tissue by β-glucuronidase activity [125]. In addition, the administration of Q3GA or IR3GA (10 mg/kg BW) to spontaneously hypertensive rats (SHRs) resulted in the progressive lowering of mean blood pressure; this effect was abolished in rats treated with a specific β-glucuronidase inhibitor [126]. Q3GA was also found to effectively inhibit NOX activity in VSMCs from both normotensive rats and SHRs, while this effect was abrogated by the addition of a β-glucuronidase inhibitor [127]. These reports indicate the importance of deconjugation reactions for the vascular function of quercetin glucuronides. A human clinical study carried out using 15 healthy volunteers by Peretz et al. [128] also demonstrated the importance of quercetin metabolite deconjugation. This double-blind, randomized, placebo-controlled trial showed that increased branchial arterial diameter following the intake of 200 mg or 400 mg quercetin aglycone for 5 h was positively correlated with the products of Q3GA levels and plasma β-glucuronidase activity. It is therefore likely that dietary quercetin exerts acute vasodilator effects in vivo because of the deconjugation of the metabolite Q3GA. Interestingly, the comparison of Q3GA, Q3′GA, Q7GA, and Q4′GA showed that Q3GA was the most responsive to activated macrophage-induced deconjugation [129].

## 8. Contribution of the Microbiota to the Anti-Atherosclerotic Effect of Quercetin Glycosides

Dietary flavonoids are only partially absorbed by epithelial cells in the small intestine and mostly pass through the intestinal tract before being excreted in feces. Until recently, the functions of unabsorbed flavonoids have been limited to the inhibition of digestive enzymes such as lipase and amylase, resulting in the regulation of lipid metabolism and blood glucose levels, respectively. However, considerable attention is now being paid to novel functions relating to the gut microbiota such as the physiological role of catabolites produced by intestinal microorganisms and the prevention of dysbiosis in the microbiota [130].

### 8.1. Function of Catabolites Produced by the Action of the Microbiota

A catabolite of quercetin in the large intestine, 3-(3-hydroxyphenyl) propionic acid, has been suggested to exert anti-inflammatory effects by inhibiting the TNFα-induced adhesion of monocytes to HAECs and suppressing the upregulation of E-selectin, without modulating the expression of ICAM-1/VCAM-1 [131]. In addition, both 3,4-dihydroxyphenyl acetic acid (3,4-DHPAA) and 3,4-dihydroxyphenyl propionic acid (3,4-DHPPA), typical chain-scission products of quercetin, suppressed the production of TNFα, IL-1β, and IL-6 in LPS-stimulated peripheral blood mononuclear cells [132]. Analysis of an acetaminophen-induced mouse model of liver injury suggested that 3,4-DHPAA protected against liver injury by activation of the Nrf-2 signaling pathway, as the resulting upregulation of phase II drug-detoxifying enzymes and antioxidant enzymes was observed to promote the nuclear translocation of Nrf-2 [133]. Another study showed that 3,4-DHPAA upregulated the expression of phase-II drug-metabolizing enzymes, thereby suppressing oxidative stress in hepatocytes [134]. Thus, this catabolite may play a key role within the digestive tract and blood circulation. Furthermore, 3,4-dihydroxytoluene (DHT), suggested to be a catabolite of rutin, inhibited the production of inflammatory cytokines by reducing NFκB signaling in LPS-activated macrophages [135]. DHT was also shown to inhibit cholesterol synthesis in primary rat hepatocytes [136], while treatment with DHT or 3,4-DHPAA decreased blood pressure in normotensive rats and SHRs [137].

### 8.2. Modulation of Gut Microbiota Profiles

Quercetin and its glycosides have been shown to exert a variety of effects on the gut microbiota. The human gut microbiota is mainly composed of the Gram-positive phyla *Firmicutes* and *Actinobacteria* and Gram-negative phyla *Bacteroidetes* and *Proteobacteria*. A study comparing the gut biota in coronary artery disease patients and healthy volunteers showed that the order *Lactobacillales* (belonging to the phylum *Firmicutes*) was increased and the phylum *Bacteroidetes* was decreased in the coronary artery disease patients [138]. This dysbiosis may be a risk factor for atherosclerotic events. Nonetheless, unabsorbed flavonoids located in the large intestine have been suggested to act as prebiotics by increasing the abundance of *Lactobacillus* and *Bifidobacterium*, resulting in the improvement of the barrier function of the large intestine [139]. In addition, quercetin aglycone (but not rutin) strongly inhibited the growth of the human intestinal microorganisms *Ruminococcus gauvreauii*, *Bacteroides galacturonicus*, and *Lactobacillus* spp. [140]. Furthermore, the administration of quercetin aglycone to LDL receptor-null mice maintained on a high-fat diet reduced the abundance of *Verrocomicrobia* and increased the abundance of *Actinobacteria*, *Cyanobacteria*, and *Firmicutes* while reducing the extent of atherosclerotic lesions [141].

Short-chain fatty acids (SCFAs) including acetic acid, propionic acid, and butyric acid, are produced primarily from the microbial fermentation of dietary fiber. These compounds are known to regulate microbial activity in the gut and participate in host health through tissue-specific mechanisms relating to gut barrier function, glucose homeostasis, and immunomodulation [142]. In a multi-reactor gastrointestinal model, rutin increased total SCFA levels following digestion for 2 h [143]. In addition, high-pressure processed onion was able to increase the abundance of beneficial colon bacteria *Bifidobacterium* spp. and *Lactobacillus* spp. and enhance the production of total SCFAs in a colon fermentation simulator [144].

Further studies indicated the protective effects exerted by quercetin on the probiotic *Lactobacillus* strain, leading to improved functionality in the host [145]. Moreover, quercetin aglycone, Q3G, and A4′G were suggested to enhance the production of anti-inflammatory substances by *Bifidobacterium adolescentis* [146,147]. Quercetin aglycone also affects the mucosal barrier covering the intestinal epithelial surface, which is vital for maintaining intestinal health [148]; in vitro studies using a small intestinal cell co-culture system showed that quercetin aglycone modulated the expression and secretion of mucin, leading to improved barrier function [149].

**Figure 7:**
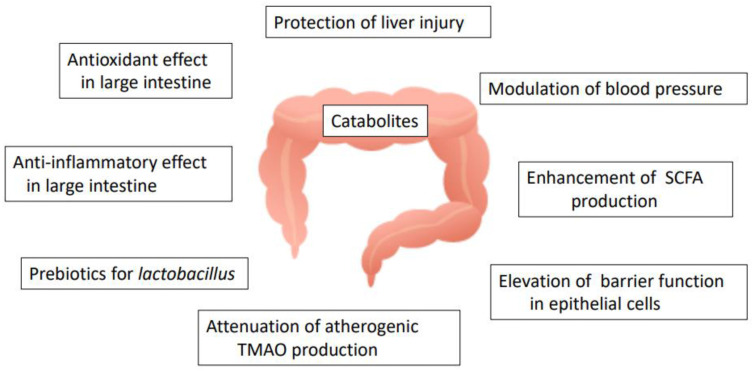
Possible role of quercetin glycosides in relation to gut microbiota activity (SCFA: short chain fatty acids; TMAO: trimethylamine oxide).

Choline (a component of phosphatidylcholine found in eggs and other common foodstuffs) and L-carnitine (found in red meat) are converted to trimethylamine (TMA) by the action of the gut microbiota and transported to the liver via the portal vein following absorption in the large intestine [150]. TMA is oxidized to trimethylamine-N-oxide (TMAO) by the liver enzyme flavin-monooxygenase-3 and then delivered into the bloodstream [151]. Interestingly, TMAO appeared to promote atherosclerosis by accelerating the induction of platelet aggregation and inflammation in blood vessels. Hu et al. [152] reported that TMAO-induced vascular dysfunction and hepatic injury were significantly attenuated by the administration of tartary buckwheat flavonoids (consisting of rutin and quercetin) to TMA-fed mice for 8 weeks at 400 or 800 mg/kg BW. In addition, quercetin 3-ramnosylgalactoside was suggested to reduce the production of TMAO from TMA by acting as a potential inhibitor of TMA-lyase [153]. The possible functions of quercetin glycosides in the large intestine are summarized in Figure 7.

## 9. Quercetin Glucosides as Senolytics

Senescent cells are defined as cells in a permanent state of growth arrest following the gradual loss of proliferative ability. Senescent cells are characterized by the spontaneous secretion of physiologically-active substances including inflammatory cytokines, growth factors, extracellular matrix metalloproteinases, and exosomes. This senescent-associated secretory phenotype (SASP) induces chronic inflammation in the surrounding tissue and contributes to the onset or exacerbation of CVD and other age-related diseases [154]. As atherosclerosis progresses with aging, senescent endothelial cells accumulate in atherosclerotic lesions and induce the infiltration of inflammatory cells by SASP [155]. The application of reagents capable of senolysis (i.e., the depletion of senescent cells), known as senolytics, is expected to aid in the development of novel treatments for CVD and other age-related diseases [156]. A powerful therapeutic strategy for the application of senolysis is the induction of senescent cell-specific apoptosis through the downregulation of Bcl-2 and Bcl-xL, members of the anti-apoptotic Bcl-2 family of proteins [157]. Quercetin aglycone was found to specifically deplete senescent cells in a study carried out in HUVECs and to act as a senolytic agent when combined with an anticancer drug (dasatinib) in a rodent study [158]. A pilot study for a human clinical trial has also been conducted, whereby a combination of dasatinib (100 mg/day) and quercetin (1000 mg/day) was administered for 3 days to nine patients with diabetic kidney disease (67.7 ± 3.1 years old) [159]. The results showed significantly decreased numbers of senescent cells in adipose tissue after 14 days and lower levels of SASP-related substances in plasma. This study suggested that high doses of quercetin may be required for the induction of senolytic effects in humans because of the low bioavailability of quercetin supplements. However, lower dosages may be sufficient when quercetin glucosides with higher bioavailability are supplied by cooked or processed foods.

## 10. Human Studies on the CVD-Preventive Effects of Quercetin Glycosides

### 10.1. Observational Studies

Multiple studies have investigated the role of quercetin glycosides in preventing CVD. For example, in their 1993 study, Hertog et al. [15] assessed flavonoid intake in a cohort of elderly men and followed them up for 5 years. The study results showed that flavonols (quercetin, kaempferol, and myricetin) and flavones (apigenin and luteolin), ingested mainly from tea, onions, and apples, were inversely associated with mortality from CHD. Flavonoids supplied in regularly consumed foods were therefore proposed to reduce the risk of death from CHD in this cohort. Analysis of sixteen cohorts in the Seven Countries Study (followed up for 25 years) also demonstrated that average flavonoid intake was inversely associated with CHD-related mortality [16]. Meanwhile, a French study showed that high consumption of flavonoid-rich foods may prevent CVD in women, but not in men [160]. Similarly, a 5-year follow-up study of Australian women aged > 75 years indicated that high levels of total flavonoid intake were associated with lower risks of all-cause and CVD mortality [161]. In 2014, a large-scale cohort study of 13,818 women in their 50 s, followed up for 15 years, discovered that greater intake of flavonoids from foods including oranges, berries, onions, and apples was associated with increased odds of healthy aging (defined as no chronic disease, no cognitive impairment, no impairment of physical function, and no limitation of mental health) [162]. The results of a large-scale cohort study on the prevention of CVD were also published recently by an Australian group. This study, which used the Danish Diet Cancer and Health Cohort (56,048 participants aged 52–60 years followed up for 23 years [18]) indicated that all-cause mortality and CVD mortality were inversely associated with dairy flavonoid intake (up to approximately 500 mg). In addition, a recent comprehensive meta-analysis of 39 prospective cohort studies on dietary flavonoids and CVD clarified that increased dietary intake of total flavonoids was linearly associated with lower risk of CVD, while quercetin intake was linearly associated with lower CHD risk [163].

Concerning flavonoid-rich foods, Galeone et al. [164] reported a case-control study of non-fatal acute myocardial infarction (AMI) in which the risk of AMI was decreased by the consumption of one or more portion of onion per week. In addition, an Iranian cohort study with a 6-year follow-up period showed that a higher habitual intake of allium vegetables (i.e., garlic and onion) was associated with reduced risk of CVD outcomes [165]. These observational studies strongly suggest the beneficial effects of onion and other quercetin glucoside-rich foods in terms of CVD prevention. Nevertheless, there may be inaccuracies in the reported levels of quercetin consumption by individuals. Metabolomic analyses using urine and blood samples may facilitate the accurate assessment of the amounts of flavonoids to which individuals have been exposed.

### 10.2. Intervention Studies

Some small-scale, double-blind, randomized, controlled studies have been carried out to assess the effects of flavonoid intake on CVD prevention. These intervention studies frequently involve flow-mediated dilatation (FMD), a non-invasive test of endothelial function that indicates the severity and extent of coronary atherosclerosis.

In a systematic review of seven trials involving healthy volunteers and patients without hypertension, Serban et al. [166] showed that supplementation with >500 mg/day of quercetin aglycone appeared to be required for the reduction of both systolic blood pressure and diastolic blood pressure. This high dosage may reflect the poor bioavailability of quercetin aglycone administered as a supplement. Another systematic review, which examined 18 randomized controlled trials concerning the effects of flavonoid-rich food products on endothelial function, showed that catechol-type flavonoids, including quercetin, improved FMD, albeit with a non-linear dose-response relationship [167]. In addition, several small-scale intervention studies have explored inflammation-related biomarkers as endpoints for the effectiveness of quercetin supplementation. For example, a randomized, placebo-controlled, crossover trial in 12 healthy men showed that oral administration of 200 mg quercetin resulted in significant increases in biomarkers of NO production (S-nitrosothiols, plasma nitrite, and urinary nitrate) and reductions in plasma and urinary endothelin concentrations [168]. Meanwhile, the administration of 150 mg quercetin/day to 93 overweight/obese subjects (aged 25–65 years) resulted in reduced systolic blood pressure after 6 weeks [169]. In this trial, quercetin intake significantly lowered the concentration of oxLDL in plasma without affecting TNF-α or C-reactive protein (CRP). Another trial, conducted in healthy male smokers, showed that supplementation with 100 mg quercetin (in capsules) for 10 weeks significantly reduced serum total cholesterol and LDL cholesterol concentrations and increased HDL cholesterol concentration; decreases in systolic and diastolic blood pressures were also observed, despite the lack of changes in IL-6 and VCAM-1 levels [170]. An intervention study that included 37 healthy hypertensive men and women aged 40–80 years receiving daily Q3G supplements (160 mg in capsules for 4 weeks) showed reduced levels of several inflammatory biomarkers, including soluble endothelial selectin, 1L-1β, and Z-score [171], although no changes in FMD or insulin resistance were seen [172]. Thus, quercetin glucosides may mediate cardioprotective effects by improving endothelial function and reducing inflammation. Nonetheless, one study indicated that acute administration of Q3G (up to 400 mg/day) to 15 healthy volunteers did not lead to any improvement in endothelial function, blood pressure, or NO production [173]. In contrast, in 25 participants with at least one CVD risk factor, increased FMD was observed 1.5 h after the administration of EMIQ (2 mg quercetin equivalent/kg BW), which is more water-soluble than Q3G [174]. Finally, a meta-analysis of 4–6 eligible randomized control studies evaluating the impact of quercetin supplementation on inflammatory biomarkers indicated that there were no relevant overall effects on peripheral CRP, IL6, or TNF-α [175]. However, subgroup analysis revealed significant reductions in circulating CRP and IL-6 in patients with diagnosed diseases and high-dose intervention. Thus, the authors concluded that the consumption of quercetin represented a promising therapeutic strategy for chronic disease management.

Regarding quercetin glucoside-rich foods, nine published works on onion intake are summarized in Table 2. In one study, continual onion consumption for 7 days did not result in any effects on platelet aggregation or other homeostatic variables, although mean plasma quercetin concentration was raised [176]. In another study, the treatment of stable type 2-diabetic patients with daily quercetin supplements (76–110 mg) provided by onion and tea for 2 weeks lowered the oxidative damage caused by hydrogen peroxide to freshly prepared lymphocyte DNA [177]. Ingestion of fried onions and fresh cherry tomatoes by non-obese normocholesterolemic female volunteers increased the resistance of lymphocyte DNA to strand breakage and decreased the levels of urinary 8-hydroxyguanosine, an oxidative stress biomarker, 4 h after ingestion of the meal [178]. In a different trial, intake of onion extracts for 30 days significantly improved FMD postprandially, while fasting FMD was not affected [179]. Moreover, in an 8-week trial, lower levels of total cholesterol and LDL cholesterol were found in patients allocated to a high-onion intervention group, compared with a low-onion control group [180]. A hypertensive subgroup of participants in a randomized 6-week control trial who received onion skin extracts showed decreases in systolic blood pressure [181]. Meanwhile, for healthy overweight and obese participants receiving daily soft capsules of onion peel extract, FMD was improved, and numbers of endothelial progenitor cells were significantly increased after 12 weeks [182]. In addition, healthy hypercholesterolemic volunteers who ingested wine containing extracts of onion exhibited substantially suppressed total cholesterol and LDL cholesterol levels after 10 weeks [183]. Nevertheless, another study showed that the daily intake of onion skin extracts did not significantly affect parameters of systemic or adipose tissue inflammation [184].

Overall, both observational and intervention studies strongly imply the beneficial cardioprotective effects of a plentiful intake of quercetin glycoside-rich foods, although definitive conclusions cannot be derived at present. Intervention trials suffer from interindividual differences in the inherent and food style-dependent bioavailability of quercetin glycosides. Large-scale, long-term intervention studies are required to precisely assess the cardioprotective effects of quercetin glycoside-rich foods. Moreover, quercetin glycoside intakes calculated from common flavonoid-rich food databases [22,23] do not show the true levels of quercetin exposure in humans. This is a problematic issue for epidemiological studies attempting to explore the relationship between the intake of quercetin glycoside-rich foods and their CVD-preventive effects. Thus, novel biomarkers that reflect real individual exposure to quercetin should be developed to facilitate the accurate assessment of the cardioprotective effects of these foods.

## 11. Conclusions and Future Perspectives

Humans consume a wide variety of polyphenols from plant foods every day. The physiological functions of polyphenols are currently of great interest in relation to their protective effects against chronic diseases [185]. Notably, 2022 saw the publication of a pioneering large-scale intervention study suggesting that the habitual intake of cacao polyphenols reduced CVD-related mortality [186]. Quercetin, a member of the monomeric flavonoid subgroup of polyphenols, accounts for a major part of the dietary flavonoids originating from vegetables. The bioactivity and bioavailability of quercetin and its related flavonoids have been extensively investigated over the last 20 years to elucidate their cardioprotective role. It should be emphasized that glycosides, rather than aglycone, are the dietary source of quercetin. Glucose-bound glucosides, which are representative of the glycosides present in onion, should be distinguished from glycosides bound to non-glucose sugars in terms of absorption from the small intestine. Nevertheless, both types of glycosides are mostly catabolized and decomposed by gut microbiota to yield aglycone and ring-scission products that can exert dietary fiber-like effects on the digestive tract and gut microbiome. In the blood, quercetin glycosides are converted to conjugated metabolites, like invading xenobiotics. Under inflammatory conditions, conjugated metabolites may be returned to their aglycone form because of the activation of deconjugation enzymes. Both conjugates and deconjugated aglycone can suppress atherosclerosis via multiple functions, such as antioxidant, anti-inflammatory, and plasma cholesterol-lowering effects, directed against a broad range of targets including endothelial cells, oxidized LDL, VSMCs, and hepatic cells. However, a comprehensive mechanism that integrates each of the functions proposed to underlie the protective effects of dietary quercetin is yet to be established. Elucidation of these cardioprotective effects will require new techniques that enable the visualization of time-dependent changes occurring at cellular and intracellular target sites following quercetin glycoside ingestion/intestinal absorption.

In general, the most abundant dietary sources of quercetin are Q3,4′diG and Q4′G, supplied from onion. Onions are considered a functional food of vegetable origin as they contain two types of bioactive compounds, sulfur-containing compounds and quercetin glycosides [187]. Here, we described the results of short-term intervention trials indicating the cardioprotective effects of onion intake (Table 2). However, the relationship between the bioefficacy and bioavailability of quercetin glycosides from onions is not yet known. A large-scale intervention trial is necessary to clarify this issue. Extensive metabolomic analysis of plasma is required, as the bioavailability of plant bioactives, including flavonoids, differs between individuals [188]. Such a study would help to confirm the role of onion quercetin glycosides as a beneficial food source capable of preventing CVD.

The therapeutic effects and safe uses of plant polyphenols have also been explored in the context of clinical treatments for CVD [189]. Interestingly, pilot studies on the senolytic properties of quercetin (i.e., the selective removal of senescent cells from the human body) have recently been initiated, using high-dose supplementation of pure quercetin aglycone in capsules or tablets. However, we have confirmed that the bioavailability of quercetin glucosides from onion or processed onion is much higher than that of pure quercetin aglycone in capsules or tablets because of the effect of the food matrix, as well as other unknown factors. Thus, onion itself could be used clinically for the prevention or treatment of CVD [190]. The high bioavailability of onion quercetin glucosides warrants their potential application as a source of dietary quercetin for CVD treatment.

## Figures and Tables

**Table 1:** Quercetin glycoside content of vegetables [22].

Compounds	Vegetables	Mean Content ± SD * (mg/100 g)	Minimum~Maximum Content (mg/100 g)
isorhamnetin 4′-O-glucoside	onion [red]	6	---
	onion [yellow]	2.89 ± 0.88	1.90~3.80
quercetin 3,4′-*O*-diglucoside	onion [red]	77.08 ± 64.44	20.22~207.49
	onion [white]	3.12 ± 2.50	0~5.00
	onion [yellow]	26.58± 31.11	11.37~111.7
	Shallot	74.62	---
quercetin 3-*O*-(6′′-malonyl-glucoside)	lettuce [green]	1.85 ± 2.79	0.03~7.33
	lettuce [red]	10.44 ±6.29	5.99~14.89
quercetin 3-*O*-garactoside (hyperoside)	lettuce [green]	0.10 ± 0.11	0~0.26
	lettuce [red]	0.14 ± 0.19	0~0.27
quercein 3-*O*-glucoside (isoquercitrin)	broccoli	1.80	---
	lettuce [green]	0.37± 0.60	0.01~1.56
	lettuce [red]	2.19 ±1.91	0.84~3.54
	onion [red]	1.80 ± 2.55	0~3.60
	onion [yellow]	0.70 ± 0.45	0~1.50
quercetin 3-*O*-glucuronide (miquelianin)	lettuce [green]	1.34 ± 1.44	0.01~3.70
	lettuce [red]	2.65 ±1.82	1.36~3.94
	green bean	0.80 ± 0.44	0.35~1.51
quercetin 3-*O*-rhamnoside (quercitrin)	lettuce [green]	0.13 ± 0.22	0~0.58
	lettuce [red]	0.85 ± 0.58	0.44~1.26
	green bean	1.99 ± 1.07	0.93~3.50
quercetin 3-*O*-rutioside (rutin)	olive [black]	45.36 ± 28.93	11.12~78.70
	tomato [cherry] whole	3.33 ± 1.77	1.79~6.61
	tomato whole	0.14 ± 0.06	0.04~0.22
	zucchini	1.32 ± 0.57	0.80~2.26
	lettuce green	0.04 ± 0.10	0~0.24
	onion [red]	0.21 ±0.06	0.17~0.27
	onion [yellow]	0.68 ±0.40	0.18~1.36
	green bean	2.49± 2.37	0.02~6.66
	asparagus	23.19 ± 10.70	0~28.65
quercetin 3-*O*-sophoroside (biamaside)	broccoli	6.5	---
Quercetin 4′-*O*-glucusode (spiraeoside)	onion [red]	38.00 ± 29.51	30.01~114.30
	onion [white]	2.25 ± 1.80	0~3.60
	onion [yellow]	21.55 ±16.86	13.77~83.03
	shallot	35.60	---

* SD: standard deviation.

**Table 2:** Intervention trials examining the anti-atherosclerotic effects of onion intake *.

Dose/Day	Period	Number of Subjects	Biomarkers Affected	Effect	Remarks	Reference
whole 220 g (114 mg quercetin equivalent)	7 days	18 healthy	platelet aggregation	no effect	randomized crossover trial	Jansen et al. [176]
whole 400 g (76~110 mg quercetin equivalent)	14 days	10 type2 diabetic	lymphocyte DNA damage	−	randomized crossover trial	Lean et al. [177]
fried onion 200 g	1 day	6 healthy	lymphocyte DNA damage 8-OHdG	− −	randomized two phase crossover trial	Boyle et al. [178]
extract 4.3 g (51 mg quercetin equivalent)	1 day	23 healthy	FMD	+	before and after study	Nakayama et al. [179]
2 whole (each 50~60 g)	8 weeks	54 overweight and obese	total cholesterol LDL	− −	randomized control parallel trial	Mamaghani et al. [180]
skin extract (162 mg quercetin equivalent)	6 weeks	31 hypertensive	LDL ambulatory blood pressure	−	randomized crossover trial	Brull et al. [181]
peel extract (100 mg quercetin equivalent)	12 weeks	72 healthy overweight and obese	FMD endothelial progenitor cells	+	randomized, double blind placebo- controlled trial	Choi et al. [182]
red wine extract 250 mL	10 weeks	23 healthy hyper-cholesterolemic	total cholesterol LDL	− −	randomized placebo-controlled trial	Chiu et al. [183]
skin extract (162 mg quercetin equivalent)	6 weeks	68 overweight to obese	serum CRP	no effect	double blind placebo-controlled crossover trial	Brull et al. [184]

* Table is modified from ref [75] with the addition of the most recent reports. (+): increase, (−): decrease.

## Data Availability

Data is contained within the article.
